# Phylogenetic Analysis of Mitochondrial Genome of Tabanidae (Diptera: Tabanidae) Reveals the Present Status of Tabanidae Classification

**DOI:** 10.3390/insects13080695

**Published:** 2022-08-03

**Authors:** Mingyue Liu, Tingting Wu, Hao Ju, Xiaoxiao Ma, Zihao Fang, Qiaocheng Chang

**Affiliations:** 1 School of Public Health, Shantou University, Shantou 515063, China; liumingyue@byau.edu.cn (M.L.); wutingting4182@126.com (T.W.); xiaoxiaoma0305@163.com (X.M.); 13169012511@163.com (Z.F.); 2 College of Animal Science and Veterinary Medicine, Heilongjiang Bayi Agricultural University, Daqing 163319, China; juhao2111@byau.edu.cn

**Keywords:** mitochondrial genome, Tabanidae, phylogenetic analyses, *Chrysops*, *Haematopota*, *Tabanus*

## Abstract

**Simple Summary:**

Tabanidae suck the blood of humans and animals, are important biological vectors for the transmission of diseases, and are of considerable economic and medical significance. However, current knowledge about the mitochondrial genome of this family is limited. Therefore, six newly completed mitochondrial genomes of four genera of Tabanidae (*Haematopota turkestanica*, *Chrysops vanderwulpi*, *Chrysops dissectus*, *Tabanus chrysurus*, *Tabanus pleskei*, and *Hybomitra* sp. species) were sequenced and analyzed. The results show that the six newly mitochondrial genomes have quite similar structures and features. Phylogeny was inferred by analyzing the 13 amino acid sequences coded by mitochondrial genes of 22 mitogenomes (all available complete mitochondrial genomes of tabanidae). Bayesian inference, maximum likelihood trees, and maximum parsimony inference analyses all showed consistent results. This study supports the concept of monophyly of all groups, ratifies the current taxonomic classification, and provides useful genetic markers for studying the molecular ecology, systematics, and population genetics of Tabanidae.

**Abstract:**

Tabanidae suck the blood of humans and animals, are important biological vectors for the transmission of diseases, and are of considerable economic and medical significance. However, current knowledge about the mitochondrial genome of this family is limited. More complete mitochondrial genomes of Tabanidae are essential for the identification and phylogeny. Therefore, this study sequenced and analyzed six complete mitochondrial (mt) genome sequences of four genera of Tabanidae for the first time. The complete mt genomes of the six new sequences are circular molecules ranging from 15,851 to 16,107 base pairs (bp) in size, with AT content ranging from 75.64 to 77.91%. The six complete mitochondrial genomes all consist of 13 protein-coding genes (PCGs), 2 ribosomal RNA genes (RRNA), 22 transfer RNA genes (tRNAs), and a control region, making a total of 37 functional subunits. ATT/ATG was the most common start codon, and the stop codon was TAA of all PCGS. All tRNA except tRNA Ser1 had a typical clover structure. Phylogeny was inferred by analyzing the 13 concatenated amino acid sequences of the 22 mt genomes. Bayesian inference, maximum-likelihood trees, and maximum-parsimony inference analyses all showed consistent results. This study supports the concept of monophyly of all genus, ratifies the current taxonomic classification, and provides effective genetic markers for molecular classification, systematics, and genetic studies of Tabanidae.

## 1. Introduction

Tabanidae is commonly known as the horsefly. Adult Tabanidae, whole metamorphosis insects, are stout, good flyers, and have scraping and licking mouthparts [1,2]. Brachycera are divided into over 20 families, with Tabanidae being one of the largest, comprising approximately 4455 species belonging to 144 genera [3,4]. Approximately 1300 species of Tabanidae belong to the genus Tabanus (subfamily Tabaninae: tribe Tabanini) [5]. Female horseflies bite and suck blood from humans and livestock, causing atopic dermatitis or anemia, resulting in weight loss in livestock. They are also important biological vectors for the transmission of several diseases, including trypanosomiasis, tularemia, and anthrax. They threaten animal husbandry and public health security, which has important significant economic and medical implications [6,7,8,9]. Different species may transmit different diseases, thus the accurate study of parasite species is important for the study of parasitic diseases, their prevention, and control methods.

Currently, Tabanidae species identification is based on morphological characteristics [10,11]. However, the body color of samples changes during storage due to the variability of physical characteristics, which leads to the false identification of tabanidae flies [12,13]. Hence, use of molecular identification methods can reduce the limitation of using only morphological features.

DNA barcoding based on mitochondrial (mt) cox 1 gene sequences has been recognized as a standard method for distinguishing various arthropod species [14,15], including tabanid flies [1,5,12,13]. Mitochondrial genome sequences are effective and reliable molecular markers for evolutionary studies of parasites due to their strict maternal inheritance, apparent lack of recombination, rapid evolutionary rate, and comparatively conserved genomic structure. It is widely used in molecular epidemiology, phylogenetic, genetics studies of different organisms, and so on [16,17]. The horsefly mitochondrial genomes has a conserved structure following the Brachycera gene number and order, consisting of 13 protein–coding genes, 2 ribosomal RNA genes, 22 transfer RNA genes, and a control region, making a total of 37 functional subunits. [18,19,20,21]. Currently, there are many methods to obtain the mitochondrial genome, such as second- and third-generation sequencing platforms suitable for molecular biological research. However, current knowledge about the mitochondrial genome of Tabanidae is limited, and species identification and classification of Tabanidae is challenging [5,12]. Thus, it is crucial to acquire more mt genome information on Tabanidae for their identification, epidemiology, and phylogeny. The identification of the horsefly species has a direct impact on related research on horseflies [22].

Therefore, the objective of this study is to use next generation sequencing (NGS) to sequence the complete mitogenome of *Ha. turkestanica*, *C. vanderwulpi*, *C. dissectus*, *T. chrysurus*, *T. pleskei*, and *Hy.* sp. In order to investigate and to provide new genetic markers for further study of taxonomic, systematics and genetics of Tabanidae, the obtained mitogenomes were compared to all available complete mitochondrial genomes of tabanidae in Brachycera.

## 2. Materials and Methods

### 2.1. Specimens and DNA Extraction

**Figure 1:**
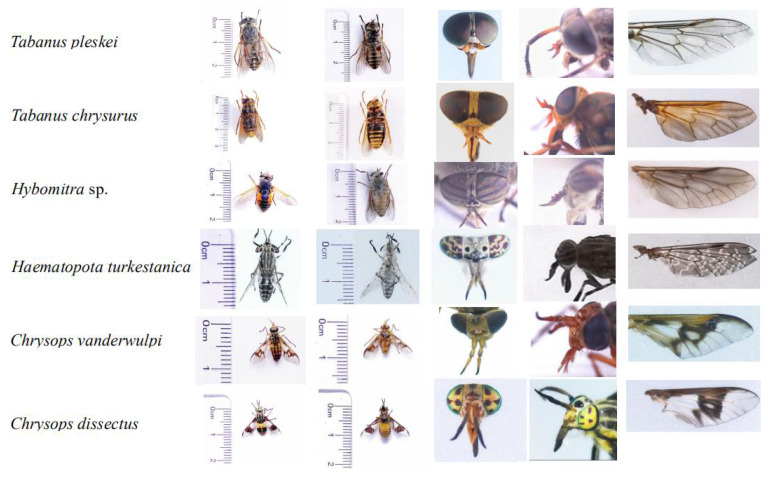
Morphological pictures of horseflies in this study, they are dorsal view of abdomen; abdomen; head; antennae; and wing.

Field expeditions in Daqing, Heilongjiang Province, China (46°71′ N, 124°86′ E) were performed to collect samples using the human attraction method. According to the external morphological characteristics, dichotomous keys were used to identify the collected representatives of Tabanidae (Figure 1) [11,23].

The samples were thoroughly washed three times in physiological saline [11,23], then fixed in 75% (*v*/*v*) ethanol and stored at −80 °C until further use. TIANamp Genomic DNA Kit (Tiangen) was used to extract insect cox 1 sequence and amplify the cox 1 sequence by PCR for molecular identification, using forward and reverse primers (5′–ATT CAA CCA ATC ATA AAG ATA TTG G–3′) and (5′–TAA ACT TCT GGA TGT CCA AAA AAT CA–3′), respectively. The final PCR reaction mix (25 μL) consisted of 18.3 μL of distilled water, 2.5 μL of 10× Ex Taq buffffer, 2 μL of dNTP mixture, 0.5 μL of each primer, 1 μL of DNA template, and 0.2 μL of Ex Taq DNA polymerase. The PCR reaction mix, in 0.2 Ml PCR tubes, was placed in a Takara TP600 thermocycler (TAKARA, Kusatsu, Japan) and the following thermocycling conditions were used: 94 °C for 5 min (initial denaturation), then 94 °C for 30 s (denaturation), 40 °C for 1 min (annealing), and 72 °C for 1 min/kb (extension) for 40 cycles, and a final extension at 72 °C for 10 min. The PCR products were sent to Sangon Biotech Company (Shanghai, China) for sequencing in both directions using the same primers.

### 2.2. Construction of the Genomic Library and Sequencing

The standard Illumina TruSeq Nano DNA LT library Preparation Guide was used to construct the required on-machine libraries. The main process was as follows (Kit: TruSeqTM DNA Sample Prep Kit, San Diego, CA, USA): (1) DNA fragmentation: Covaris is used to interrupt DNA and make it fragmented, and the target fragment is purified and sorted by magnetic beads; (2) DNA double–end Repair: the DNA fragment with protruding End was repaired by the joint action of 3‘–5’ exonuclease and polymerase in End Repair Mix; (3) Introduction of “A” base at the 3‘end: introduction of A single base “A” at the 3’ end of the repaired and flat DNA fragment; The 3‘end of the joint contains A single base “T”, so as to ensure that the DNA fragment and the joint can be connected by “A” and “T” complementary pairing, and to prevent the DNA insertion fragments from connecting to each other in the process of connecting the DNA fragment; (4) Connector connection: under the action of ligase, the connector containing the tag is incubated with the DNA fragment to make it connected; (5) Purification of ligation products: magnetic beads purify ligation products to remove free and self–ligation sequence; (6) Verification library: quantitative library using Pico Green; Agilent Bioanalyzer 2100 (State of California) was used for quality control of PCR-enriched fragments to verify the size and distribution of DNA library fragments; (7) Homogenized and mixed library: multiplexed DNA libraries (multiplexed DNA libraries) homogenized to 10 nM after equal volume mixing; and (8) Sequencing: the mixed library (10 nM) was gradually diluted and quantified to 4–5 pM before computer sequencing. The raw data generated were transferred to a computer workstation for analysis and genome characterization. In order to obtain high–quality sequences, FastQC V.0.11.9 [24] software was used to verify the quality index of the data obtained, and Trim Galore V.0.6.5 [25] software was used to remove the adaptation sequences. Finally, Fast QC software was used for further quality inspection and data verification.

### 2.3. Genomic Assembly

The obtained high-quality second-generation sequencing data were assembled from scratch using A5–MISeq V20150522 [26] and SP Adesv3.9.0 [27] software, respectively. For collinearity analysis, Mummerv3.1 software [28] was used to complete any Spaces between contigs. The final mitochondrial gene sequences was obtained by Pilon V1.18 software [29]. The assembled mt genomes were compared with the same genus in the genebank (*Haematopota vexativa* Genbank ID: NC059934.1, *Tabanus haysi* Genbank ID: MW182417.1, *Atylotus miser* Genbank ID: NC030000.1, *Cydistomyia duplonotata* Genbank ID: DQ866052.1, *Tabanus formosiensis* Genbank ID: MW182416.1, and *Tabanus amaenus* Genbank ID: MW182415.1) and were checked manually.

### 2.4. Annotation and Bioinformatics Analysis

The complete mitochondrial genome sequences obtained by splicing were uploaded to MITOS Web for function annotation (http://mitos2.Bioinf.unileipzig.de/index.py accessed on 1 July 2022) [30]. TRNAscan–SE was used to identify the secondary structure of tRNA (http://lowelab.ucsc.edu/tRNAscan–SE accessed on 1 July 2022) [31]. DNAStar (V.5.0) was used to calculate the boundaries between genes of A + T, G + C and content, AT–skew = (A − T)/(A + T) and GC–skew = (G − C)/(G + C) [32]. An online open reading frame finder was used to analyze and translate PCGs (https://www.ncbi.nlm.nih.gov/orffinder/ accessed on 1 July 2022). The relative synonymous codon usage (RSCU) of PCGs was determined using the invertebrate mitochondrial genetic code of MEGA X [33]. Comparisons in gene lengths and nucleotides were made between *Ha. turkestanica*, *C. vanderwulpi*, *C. dissectus*, *T. chrysurus*, *T. pleskei*, and *Hy*. sp., all of which belong to the Tabanidae family. Differences in the nucleotide and amino acid sequences were calculated using the MEGAX software. Sliding window analysis was also used to estimate nucleotide diversity (π) between the mitogenomes of Tabanidae species by overlapping at 25 bp intervals for every 200 bp and π was drew at the midpoint position. DnaSP software (V.6) was used to compare the proportion of nonsynonymous (dN) and synonymous (dS) substitutions (dN/dS) in the obtained sequences [34].

### 2.5. Phylogenetics Analysis

Concatenated amino acid sequences of the complete *Ha. turkestanica*, *C. vanderwulpi*, *C. dissectus*, *T. chrysurus*, *T. pleskei*, and *Hy.* sp. mt genome were aligned with the corresponding amino acid sequences of 15 horseflies from a suborder all available in GenBank, with *Anopheles sacharovi* (MZ382545) as the outgroup. The MAFFT algorithm was used to align all 13 PCGs of each sequence obtained in this study and GenBank database [35].

Phylogenetic relationships between the analyzed species were reconstructed using three methods: BI, MP, andML. MrBayes 3.1 was used to reconstruct the BI tree, and four independent Markov chain runs were performed for 1,000,000 metropolis–coupled (MCMC) generations, sampling a tree every 100 generations. The first 25% (2500) of the generated trees were omitted as burn-in, with the remaining trees being used to calculate Bayesian posterior probabilities. Phylogenetic trees were visualized using the FigTree software. MP methods were performed using the Fitch criterion (1000 bootstrap replicates) within PAUP 4.0 Beta 10. The ML methods were performed using MEGA X software (https://www.megasoftware.net accessed on 1 July 2022), and bootstrapping was performed with 1000 replicates. Phylograms were drawn using FigTree (v. 1.42) [36].

The cox 1 sequences of the six horseflies in this study were compared with 31 horseflies of five genera of Tabanidae in GenBank, using *Rhamphomyia insignis* as the outgroup (KT225299.1). A phylogenetic evolutionary tree was constructed using the ML method, as described above.

## 3. Results and Discussion

### 3.1. Acquisition of Mitochondrion cox1 Genes

**Figure 2:**
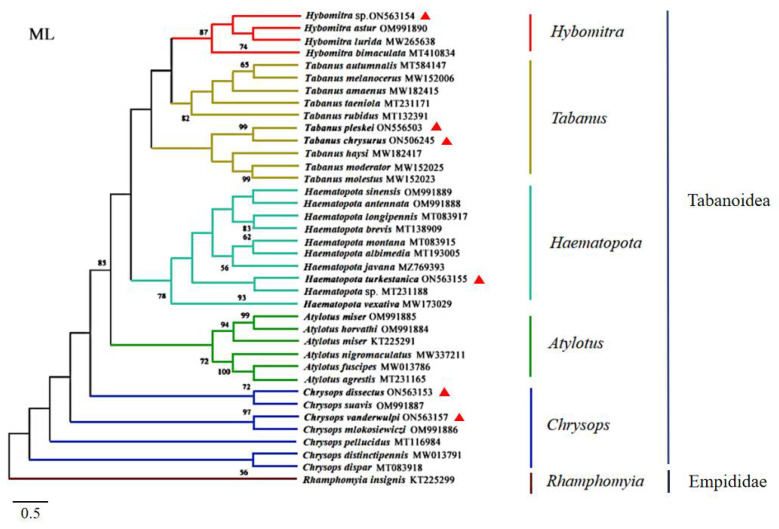
Phylogenetic reconstruction of 38 cox 1 sequences was performed using the maximum likelihood analysis. The species sequenced in this study are indicated with red triangles.

The specimens were identified as *Ha. turkestanica*, *C. vanderwulpi*, *C. dissectus*, *T. chrysurus*, *T. pleskei*, and *Hy*. sp., which was verified by morphological characteristics. *Hy.* sp. identification was not species-specific as only the genera was identified. The amplified cox1 sequences were observed to be 93.5%, 93.8%, 96.2%, 95.62%, 96.48%, and 96.15% identical to the sequences of species available in GenBank (MT231188, OM991886, OM991887, NC062705, NC062705, and MT410834), respectively. The cox 1 sequences of the six horseflies in this study were compared with 31 horseflies of five genera of Tabanidae in GenBank, using *Rhamphomyia insignis* as the outgroup (KT225299.1). A phylogenetic tree was constructed using an ML analytical approach. The results of the phylogenetic trees were consistent with those of the six branches. In this study, *Hy.* sp. was noted to be in the same branch as *Hybomitra astur*; *Hybomitra lurida*, and *Hybomitra bimaculata*, all belonging to the genus *Hybomitra*. Furthermore, *T. pleskei* and *T. chrysurus* were observed to belong to the genus Tabanus and *Ha. turkestanica* was observed in the genus *Haematopota*. The six horseflies of the genus *Atylotus* were noted to belong to the same branch and both *C. dissectus* and *C. vanderwulpi* were located in the same branch as the horseflies of the genus *Chrysops* (Figure 2). The results of cox 1 sequence-based evolution analysis were consistent with the results of homology and morphology analyses, indicating that our identification methods are accurate.

### 3.2. mtDNA Features of Six Horseflies in This Study

**Figure 3:**
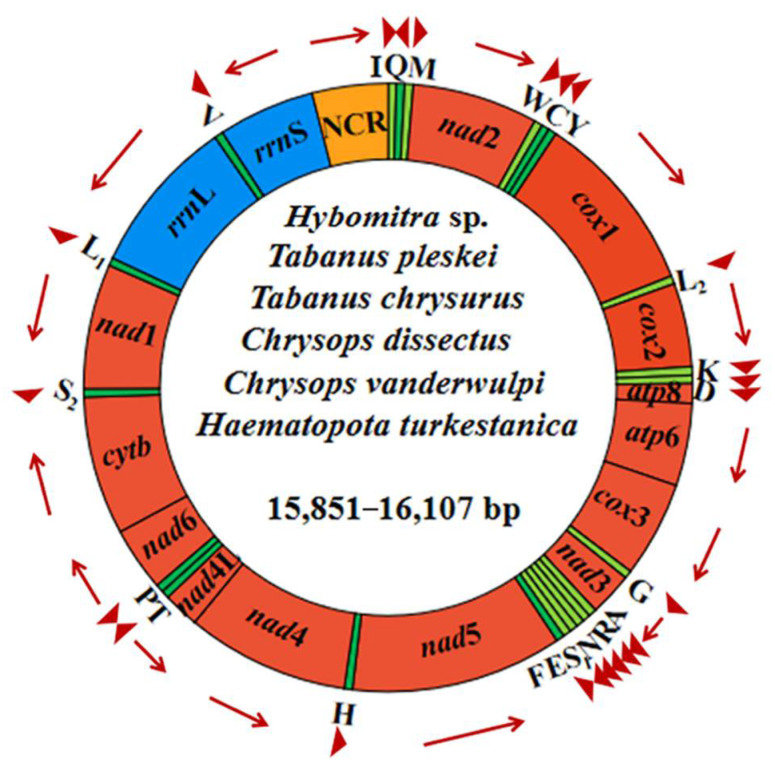
Structural representations of the six newly sequenced horseflies mitogenomes. The circle represents the arrangement of PCGs, tRNAs, and rRNAs. Each tRNA is identified by a unique letter abbreviation. ‘NCR’ indicates the non-coding region.

Complete mitogenomes of *Ha. turkestanica*, *C. vanderwulpi*, *C. dissectus*, *T. chrysurus*, *T. pleskei*, and *Hy.* sp. ranged from 15,851–16,107 bp in length, and were arranged in a classic double-stranded circular DNA molecules. The complete mt genome of *Hy.* sp. obtained in this study is the first of the genus *Hybomitra*. Detailed annotations of the mt genomes of the six newly sequenced, including the position and length of each gene and direction of the sequence, are reported in Figure 3.

All mitogenomes comprised 13 PCGs (cox 1–3, nad 1–6, nad 4L, atp 6, atp 8, and cyt b), 2 rRNA (rrn L and rrn S), 22 tRNAs and a control region, a total of 37 functional subunits, organized along the N (forward) and J (reverse) strands (Table 1). It has the same structure as other horseflies. Each of the six newly sequenced mitochondrial genomes had one or only one control region, ranging in length from 946 to 1122 bp. The control region contains a large number of AT–rich regions and repeats, which have been shown to be the main reason for the failure of PCR amplification and Sanger sequencing of complete split genomes in invertebrates [37,38].

The sequences of this study were compared with those of Diptera families, and the results showed that the six newly sequenced horseflies had the same gene order as Tanbanidae, Athericidae, and Rhagionidae species are all from Brachycera, but were different from the Culicidae, Sciaridae, and Trichoceridae species all from Nematocera. Tabanidae mitochondrial genomes are conserved and characterized by the sequence in Diptera. In their mt genome, 23 genes were located on the plus strand, and the remaining 14 genes were located on the minus strand (Figure S1) [18,19,20,21].

**Figure 4:**
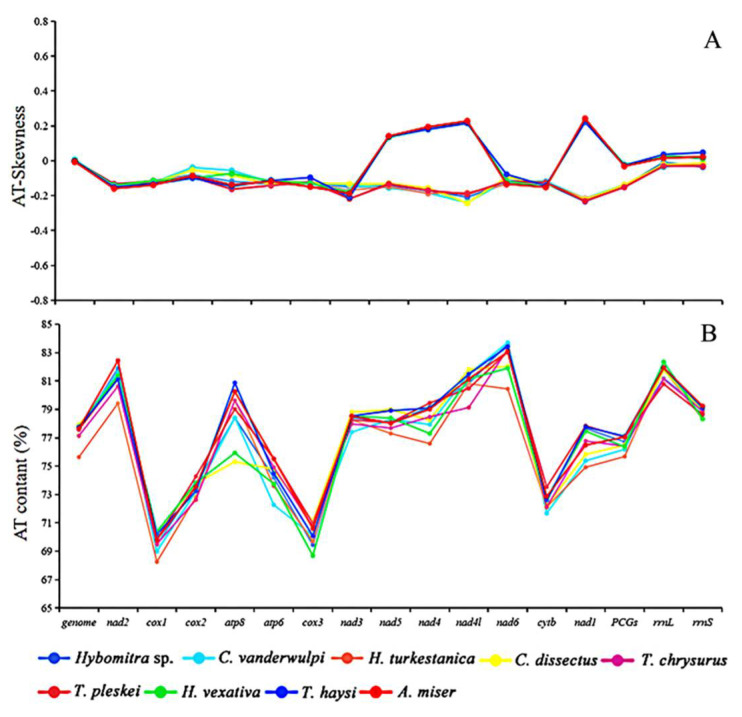
Information on AT content and skews of the investigated mitogenomes. (**A**) AT–skews (%). (**B**) AT content.

In this study, the AT content of the mt mitochondrial genomes were ranging from 75.64 to 77.91%, which are similar to the putative characteristics of previously reported Tabanidae mt genomes, including *Ha. vexativa* 77.75%, *T. haysi* 77.77%, and *A. miser* 77.65% [39,40,41]. The AT skew of these newly sequenced mitogenomes ranged from −0.01 (*Ha. turkestanica*) to 0.01 (*C. vanderwulpi*), and the GC-skew ranged from −0.21 (*Ha. turkestanica*) to −0.17 (Figure 4). There is a clear stand bias exists in their mt genomes, as is other Dipteran mt genomes [39].

### 3.3. Characteristics of Protein–Coding Genes (PCGs)

The genomes had 13 PCGs, with lengths ranging from 11,043 to 11,289 bp (AT% content ranging from 75.68 to 77.03%). The 13 subunits that are commonly reported in other Dipteran mitogenomes are: nad 5 > cox 1 > nad 4 > cyt b > nad 2 > nad 1 > cox 3 > cox 2 > atp 6 > nad 6 > nad 3 > nad 4L > atp 8 [18,40]. Of all the obtained mitochondrial genomes, 9 PCGs showed transcription on the plus strand (N): nad 2, cox 1, cox 2, atp 8, atp 6, cox 3, nad 3, nad 6, and cyt b, with the remaining genes exhibiting a sense of transcription on the reverse strand (J): nad 5, nad 4, nad 4L, and nad 1.

All 13 PCGs (except *Hy.* sp. and *C. vanderwulpi* for nad 5 with GTG) used ATN as the start codon, with ATG being the most frequently used (cox 2, atp 6, cox 3, nad 4, nad 4L, and cyt b), followed by ATT (nad 2, cox1, atp 8, and nad 3). All genes used TAA as the standard stop codon (except *T. chrysurus* and *T. pleskei* for nad 5, *T. chrysurus* for cytb, *Ha. turkestanica* for cox3 and nad 6 using TAG as stop codon) (Table 1). The canonical start codons most commonly used for invertebrate mitogenomes are ATN, TTG, GTT, and GTG. Generally, horsefly PCGs use ATN as the start codon, whereas the nad 5 gene in some species use GTG as the start codon, which is considered common across various organisms [17,42,43].

**Figure 5:**
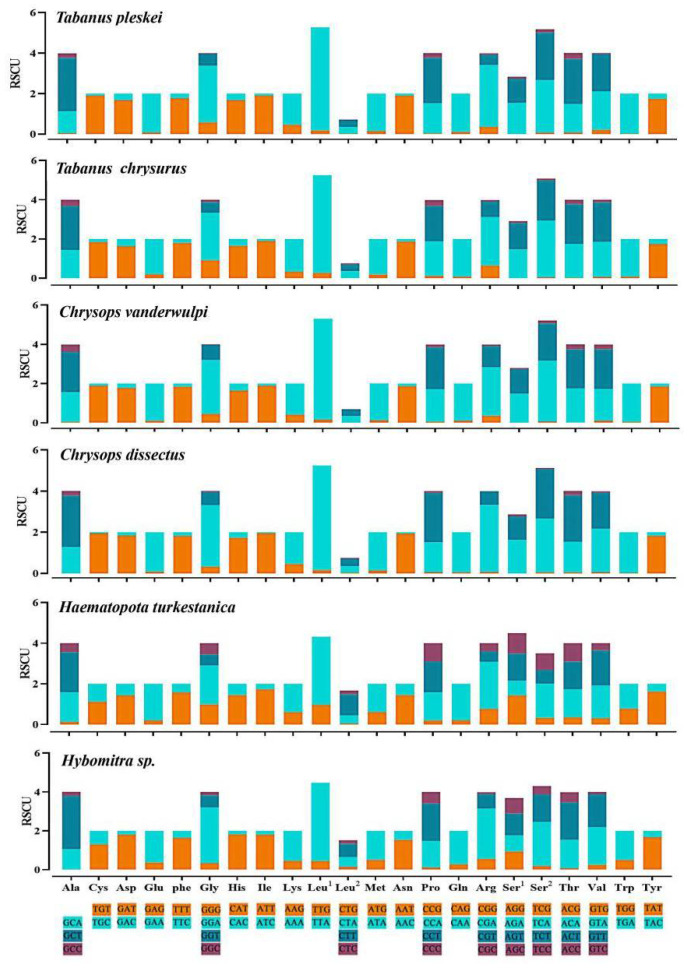
Relative synonymous codon usage (RSCU) of the obtained mitogenomes. RSCU values are represented on the y-axis, and the Aedini tride codons of their respective amino acids represented on the *x*-axis.

The overall codon usage, RSCU, used in all 13 PCGs, was presented. The most common amino acids were leucine (8.98–16.14%), serine (9.03–13.25%), and phenylalanine (8.92–12.76%), and the amino acids with low representation were His (1.08–2.20%), Arg (0.84–1.49%), and Asp (0.97–1.94%). The relative synonymous codon usage (RSCU) showed similar codon usage to the CUB analysis (Figure 5). Codons ending with A or U were more frequently used than codons ending with CG or GC, which is a common feature noted in several dipteran insects [41].

### 3.4. Analysis of the RNA (2 rRNAs and 22 tRNAs)

The location of the two ribosomal RNA genes (rRNAs, rrn L and rrn S) are conserved in all mitogenomes, with rrn L being flanked by trn L1 and trn V, and rrn S being flanked by the trn V- and AT-rich regions. The AT content was similar, despite almost all species belonging to different genera. Individually, the variation in length of the six rRNAs was minimal. The large subunit of mt RNA of the six horseflies varied from 1293 bp in *Ha. vexativa* to 1332 bp in *T. pleskei*, and the small subunit was 789 bp long in *Ha. vexativa* and 796 bp in *Tabanus* spp. mitogenome. These values were similar to those of previous horsefly mitogenome studies.

The total length of the 22 tRNAs ranged from 2076 to 2444 bp in the six newly sequenced mt genomes, and the individual gene lengths varied from 64 to 72 bp (Table 1). All six horseflies characteristically lacked a DHU arm. The lack of a DHU arm was observed in trnS1 of several other metazoans [44], including tabanid flies [23]. The most common nucleotide mismatch was G–U, followed by U–U, which plays an important role in maintaining the stability of tRNA secondary structure (Figure S2).

### 3.5. Evolutive Analysis

**Figure 6:**
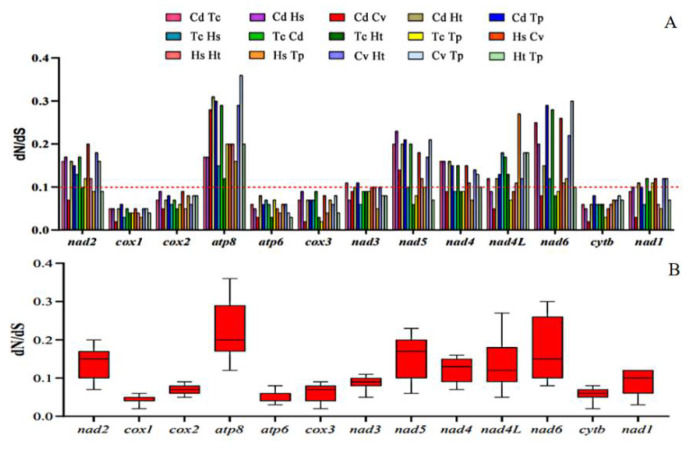
Proportions between rates of non-synonymous (dN) and synonymous (dS) nucleotide substitutions (dN/dS). (**A**) Bar chart of the pairwise proportions of dN/dS for each of the mitochondrial subunits of the investigated species. (**B**) Box chart illustrating the averages for pairwise proportions of dN/dS for each of the mitochondrial subunits of the investigated species. The dN/dS ratios are plotted on the *y*-axis, and the PCGs are plotted on the *x*-axis.

In genetics, dN/dS represents the ratio between non-synonymous (dN) and synonymous replacement rates (dS). This ratio determines whether there is selection pressure on the protein-coding gene. The corresponding sequences of each PCG in the studied species were paired based on observations of non-synonymous and synonymous replacement rates (dN/dS). The results are shown in two images. The results obtained indicated that the different regions evaluated are evolving globally under the effect of negative pressure, with dN/dS values < 1 and with ratios ranging from cox 1 (0.02 to 0.06) to atp 8 (0.12 to 0.36). Thus, the order of influence of evolutionary pressure was depicted according to the averages obtained: cox 1 < atp 6 < cyt b < cox 3 < cox 2 < nad 3 < nad 1 < nad 4 < nad 4L < nad 2 < nad 5 < nad 6 < atp 8 (Figure 6). In addition, as reported in other studies, PCGs belonging to mitochondrial complexes III and IV had strong purification pressures and PCGs belonging to the complex I region were weaker. Although the dN/dS rates of atp 8 and nad 6 were < 1, there were signs of weak purification pressure and transient positive evolutionary pressure [16,45,46].

**Figure 7:**
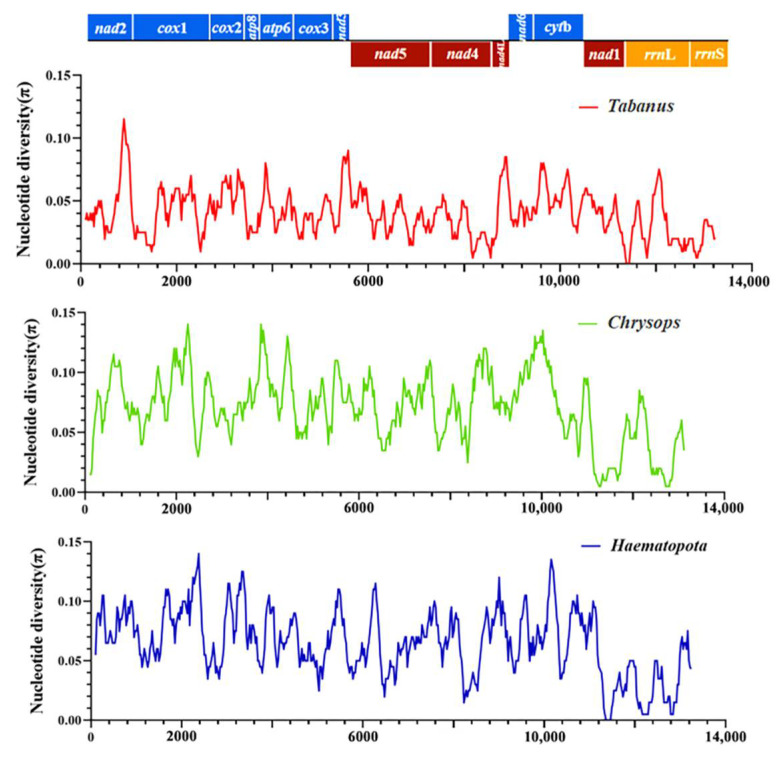
Nucleotide diversity (π) among the obtained mitogenomes in this study.

Three additional analyses were performed to assess π among the mitochondrial sequences obtained in this study and other horsefly sequences of Tabanidae. Only one mitochondrial whole genome sequence was found in *Hybomitra*, and thus the nucleotide diversity analysis of *Hybomitra* could not be performed. The first analysis evaluated the π among mitochondrial sequences of horseflies belonging to the genera of *Tabanus*, the second analysis evaluated the π belonging to the *Chrysops*, and the third analysis evaluated the π belonging to the *Haematopota*. Figure 7 showed the values of π, considering the groups of evaluated sequences. The π values ranged from 0.01 to 0.12 in *Tabanus* (red line), 0.01 to 0.12 in *Chrysops* (green line); 0.00 to 0.11 in *Haematopota* (blue line) (Figure 7). The concatenated sequences revealed that the lowest nucleotide diversity was observed in nad 1 in *Tabanus*; *Chrysops* and *Haematopota*. The nad 2, nad 6, and cytb showed higher nucleotide diversity in all three data sets [41].

The red lines indicate π values based on the evaluation of sequences from *Tabanus* genu, including *Tabanus chrysurus* and *Tabanus pleskei*. Green lines indicate π values based on the evaluation of sequences from the genus *Chrysops*, including *Chrysops dissectus* and *Chrysops vanderwulpi*. Blue lines indicate π values based on the evaluation of sequences from *Haematopota* genus, including *Haematopota turkestanica* and *Haematopota vexativa*. Values were calculated from a 200–bp sliding window analysis of 25 bp steps and plotted on the *y*-axis, and the length of the sequence was plotted on the *x*-axis.

### 3.6. Phylogenetic Analyses

**Figure 8:**
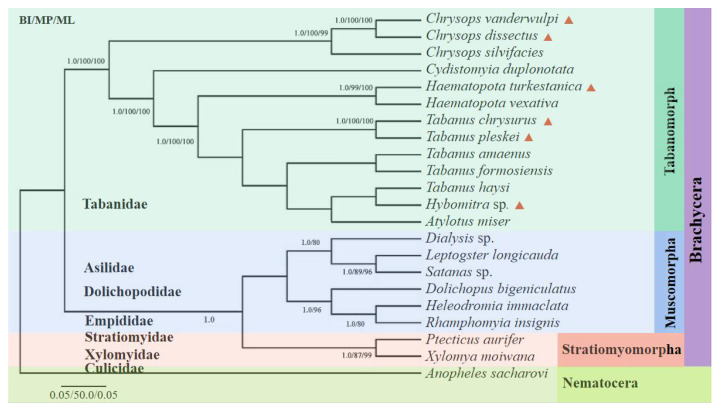
Phylogenetic reconstruction by analyzing the 13 PCGs concatenated amino acid sequences of 22 mitogenomes using Bayesian inference, maximum likelihood and maximum parsimony analysis. The species sequenced in this study were marked with red triangles. Values less than 80 are not displayed.

Phylogenetic analyses of the amino acid concatenated coding regions were aligned using the 13 PCGs from 22 taxa (six from this study, the remaining from the GenBank database) using three analytical approaches (BI, MP, and ML). All the methods produced nearly identical tree topologies (Figure 8). A monophyletic large group included 21 taxa corresponding to the suborder Brachycera, with *Anopheles sacharovi* (Diptera: Culicidae) serving as the outgroup. The suborder Brachycera presented a topology with two well-supported clades, with the top clade corresponding to the subfamilies Tabanomorpha and the lower clades corresponding to Muscomorpha and Stratiomyomorpha. The Tabanomorpha clade, contained 13 species (including the six gadflies sequenced in this study), and five subclades, representing the genera *Chrysops*, *Haematopota*, *Tabanus*, *Hybomitra*, and *Atylotus*, were recovered as a sister group to the Tabanidae tribe, as previously indicated. The phylogenetic trees revealed that *C. dissectus* and *C. vanderwulpi* belonged to the genus *Chrysops* in the same clades as *Chrysops silvifacies*; *Ha. turkestanica* and *Ha. vexativa* belonged to the genus *Haematopota* in the same clade; *T. chrysurus*, in the same clade as *T. pleskei*, belonged to the genus *Tabanus*. *Hy.* sp., and *T. haysi* were closely related in the same clade, followed by *A. miser.*

Fu et al. (2021) reported that ML and BI phylogenies showed nonmonophyletic relationships among species of *Tabanus* and/or *Atylotus*, where *A.* *miser* clustered with *T. formosiensis*, while *Tabanus* did not form a single clade. Previous phylogenetic analyses of the nuclear and mitochondrial genomes based on conventional barcoding have also suggested paraphyletism in the genera *Tabanus* and/or *Atylotus* [1,5,47,48].

The evolutionary tree constructed in this study showed that *Hy.* sp. and *T. haysi* were in the same branch. The evolutionary tree constructed with the cox 1 sequence showed that *Hy.* sp. was in the same branch as *Hybomitra astur*; *Hybomitra lurida*, and *Hybomitra bimaculata*, and they all belong to the genus *Hybomitra*. However, *T. haysi* was in the genus *Tabanus*, with each genus having its own separate branch, which was consistent with the method of using cox 1 as molecular marker.

Therefore, it is considered that such classification results can be formed because there was only one sequence of *Hybomitra* in this study, which is the first reported for the genera. Therefore, *Hy.* sp. was in the same clade as *T. haysi*, suggesting that phylogenetic analysis using only 21 protein-coding sequences of these species is not representative of the 4455 clades of these species. The evolutionary relationship between the genera *Atylotus* and *Hybomitra* within Tabanidae remains unclear, owing to the scarcity of complete mitogenome sequences. Due to its higher mutation rate, mt DNA is generally more prone to saturation than the nuclear genome. Thus, in order for a more accurate and comprehensive analysis of the classification and evolution of horseflies, it is necessary to sequence more mitogenomes of horseflies and include them in future analyses to solve the problem of horsefly classification, as complete mitochondrial genome sequences have been shown to resolve the phylogenetic relationships of many other surface parasites [49,50,51,52].

## 4. Conclusions

In this study, we first determined the complete mitochondrial genomes of six horseflies, and secondly, the mitochondrial genomes characteristics and phylogenetic analysis of six newly sequenced were carried out. All mitogenomes evaluated were similar to the mtDNA molecular pattern for the Tabanidae family: 37 subunits were subdivided into 13 PCGs, 22 tRNAs, 2 rRNAs, and a control region. All tRNAs had a typical leaf clover structure, except tRNA Ser 1. The three analysis methods, BI, MP, and ML, yielded similar topologies. Our mitogenomes phylogenetic analysis supports the paraphyly of genus *Tabanus*; *Chrysops* and *Haematopota*. Only 21 sequences of these species is not representative of the whole family Tabanidae. Due to a lack of complete genome sequence, *Hybomitra* and *Atylotus* genera evolutionary relationship are unclear. Thus, in order for a more accurate and comprehensive analysis of the classification and evolution of horseflies, it is necessary to sequence more mitogenomes of horseflies to solve the problem of horsefly classification to enrich the knowledge of their molecular aspects. These findings provide new genetic markers for further studies of taxonomic, systematics, and genetics of Tabanidae.

## Figures and Tables

**Table 1:** Mitochondrial genome organization of *Hybomitra* sp., *Haematopota turkestanica*, *Chrysops vanderwulpi*, *Chrysops dissectus*, *Tabanus chrysurus*, and *Tabanus pleskei*.

Genes	Strand	Position and nt Sequence Length (bp)						Initiation Codon	Stop Codon	Anticodon
		*Hybomitra* sp.	*Haematopota* *turkestanica*	*Chrysops vanderwulpi*	*Chrysops dissectus*	*Tabanus chrysurus*	*Tabanus* *pleskei*			
*trn*I	N	1–66 (66)	1–66 (66)	1–67 (67)	1–67 (67)	1–66 (66)	1–66 (66)			GAT
*trn*Q	J	68–136 (69)	64–132 (69)	65–133 (69)	65–133 (69)	67–135 (69)	67–135 (69)			TTG
*trn*M	N	149–218 (70)	141–210 (70)	152–220 (69)	138–206 (69)	146–215 (70)	146–215 (70)			CAT
*nad*2	N	219–1250 (1032)	211–1242 (1032)	221–1252 (1032)	207–1238 (1032)	216–1247 (1032)	216–1247 (1032)	ATT	TAA	
*trn*W	N	1249–1317 (69)	1241–1309 (69)	1256–1324 (69)	1242–1310 (69)	1247–1316 (70)	1247–1316 (70)			TCA
*trn*C	J	1310–1373 (64)	1302–1369 (68)	1317–1379 (63)	1303–1367 (65)	1309–1373 (65)	1309–1373 (65)			GCA
*trn*Y	J	1376–1442 (67)	1375–1441 (67)	1389–1456 (68)	1377–1444 (68)	1375–1441 (67)	1375–1441 (67)			GTA
*cox*1	N	1477–2982 (1506)	1476–2981 (1506)	1491–2900 (1500)	1479–2978 (1500)	1476–2981 (1506)	1476–2981 (1506)	ATT	TAA	
*trn*L2	N	2978–3043 (66)	2977–3042 (66)	2995–3060 (66)	2983–3048 (66)	2977–3042 (66)	2977–3042 (66)			TAA
*cox*2	N	3045–3732 (688)	3044–3731 (688)	3062–3748 (687)	3050–3736 (687)	3044–3731 (688)	3044–3731 (688)	ATG	T(AA)	
*trn*K	N	3733–3803 (71)	3732–3802 (71)	3750–3820 (71)	3738–3808 (71)	3732–3802 (71)	3732–3802 (71)			CTT
*trn*D	N	3805–3871 (67)	3805–3871 (67)	3820–3887 (68)	3813–3881 (69)	3811–3877 (67)	3807–3873 (67)			GTC
*atp*8	N	3872–4033 (162)	3872–4033 (162)	3888–4049 (162)	3882–4043 (162)	3878–4039 (162)	3874–4035 (162)	ATT/ ATC	TAA	
*atp*6	N	4027–4704 (678)	4027–4704 (678)	4043–4720 (678)	4037–4714 (678)	4033–4710 (678)	4029–4706 (678)	ATG	TAA	
*cox*3	N	4704–5492 (789)	4704–5492 (789)	4720–5508 (789)	4714–5502 (789)	4710–5498 (789)	4706–5494 (789)	ATG	TAA/ TAG	
*trn*G	N	5495–5560 (66)	5495–5560 (66)	5510–5575 (66)	5504–5569 (66)	5501–5566 (66)	5497–5562 (66)			TCC
*nad*3	N	5558–5914 (357)	5561–5914 (354)	5576–5929 (354)	5570–5923 (354)	5567–5920 (354)	5563–5916 (354)	ATA/ ATT	TAA	
*trn*A	N	5918–5984 (67)	5918–5984 (67)	5935–6001 (67)	5929–5995 (67)	5924–5990 (67)	5920–5986 (67)			TGC
*trn*R	N	5984–6047 (64)	5984–6047 (64)	6001–6065 (65)	5995–6059 (65)	5990–6053 (64)	5986–6049 (64)			TCG
*trn*N	N	6050–6115 (66)	6050–6115 (66)	6066–6134 (69)	6062–6127 (66)	6056–6121 (66)	6052–6118 (67)			GTT
*trn*S1	N	6116–6182 (67)	6116–6182 (67)	6135–6201 (67)	6128–6194 (67)	6122–6188 (67)	6119–6185 (67)			GCT
*trn*E	N	6183–6249 (67)	6183–6249 (67)	6203–6268 (66)	6198–6263 (66)	6189–6255 (67)	6186–6252 (67)			TTC
*trn*F	J	6266–6333 (68)	6266–6333 (68)	6285–6351 (67)	6280–6347 (68)	6272–6339 (68)	6269–6336 (68)			GAA
*nad*5	J	6334–8068 (1735)	6334–8068 (1735)	6351–8087 (1737)	6347–8083 (1737)	6340–8074 (1735)	6337–8071 (1735)	GTG/ ATT	T(AA)	
*trn*H	J	8069–8135 (67)	8069–8135 (67)	8088–8154 (67)	8084–8150 (67)	8075–8141 (67)	8072–8138 (67)			GTG
*nad*4	J	8136–9474 (1339)	8135–9475 (1341)	8155–9493 (1339)	8151–9489(1339)	8141–9480 (1340)	8138–9477 (1340)	ATG	T(AA)	
*nad*4L	J	9468–9764 (297)	9469–9765 (297)	9487–9783 (297)	9483–9779 (297)	9474–9770 (297)	9471–9767 (297)	ATG	TAA	
*trn*T	N	9767–9831 (65)	9768–9832 (65)	9786–9851 (66)	9782–9847 (66)	9773–9837 (65)	9770–9834 (65)			TGT
*trn*P	J	9832–9897 (66)	9833–9898 (66)	9852–9917 (66)	9848–9913 (66)	9838–9903 (66)	9835–9900 (66)			TGG
*nad*6	N	9900–10,424 (525)	9901–10,425 (525)	9920–10,441 (522)	9916–10,440 (525)	9906–10,430 (525)	9903–10,427 (525)	ATT/ ATA	TAA	
*cyt*b	N	10,429–11,565 (1137)	10,430–11,566 (1137)	10,449–11,585 (1137)	10,448–11,584 (1137)	10,434–11,570 (1137)	10,431–11,567 (1137)	ATG	TAG/ TAA	
*trn*S2	N	11,564–11,631 (68)	11,565–11,632 (68)	11,599–11,668 (70)	11,595–11,664 (70)	11,569–11,636 (68)	11,570–11,637 (68)			TGA/TCA
*nad*1	J	11,638–12,595 (958)	11,639–12,596 (958)	11,601–12,632 (1032)	11,672–12,628(957)	11,643–12,600 (958)	11,644–12,601 (958)	ATA/ TTG/ ATG	TAA	
*trn*L1	J	12,597–12,661 (65)	12,598–12,662 (65)	12,634–12,697 (64)	12,630–12,693 (64)	12,602–12,665 (64)	12,603–12,666 (64)			TAG/TGA
*rrn*L	J	12,675–13,970 (1296)	12,675–13,967 (1293)	12,675–14,002 (1328)	12,671–13,997 (1327)	12,677–13,974 (1298)	12,644–13,975 (1332)			
*trn*V	J	13,995–14,066 (72)	13,995–14,066 (72)	14,030–14,101 (72)	14,025–14,096 (72)	13,999–14,070 (72)	14,000–14,071 (72)			TAC
*rrn*S	J	14,066–14,859 (794)	14,066–14,863 (789)	14,101–14,895 (795)	14,096–14,887 (792)	14,070–14,865 (796)	14,071–14,866 (796)			
AT	N	14,860–15,851 (992)	14,864–15,893 (1030)	14,896–16,017 (1122)	14,888–15,833 (946)	14,866–15,853 (988)	14,867–15,856 (990)			

## Data Availability

All data generated during this study are available as tables and figures in the published article and its Supplementary Materials. The GenBank database accession numbers for the six mitochondrial genomes sequenced in this study are: ON675578 (*Haematopota turkestanica*), ON660873 (*Chrysops vanderwulpi*), ON675579 (*Chrysops dissectus*), ON660872 (*Tabanus chrysurus*), ON660871 (*Tabanus pleskei*), and ON653032 (*Hybomitra* sp.).

## Supplementary Materials

The following are available online at https://www.mdpi.com/article/10.3390/insects13080695/s1. Figure S1: Comparison of linearized mitogenomes of Diptera species. The tRNA genes are shown by a single-letter code of corresponding amino acids. Newly sequenced mitogenomes are indicated in bold. Asterisks indicate the incomplete mitogenomes. Figure S2: Secondary structures of tRNAs of *Hybomitra* sp.; *Chrysops vanderwulpi*; *Haematopota turkestanica*; *Chrysops dissectus*; *Tabanus chrysurus*; and *Tabanus pleskei*.
