# Supplementary figures and images for: Phylogenetic Analysis of Mitochondrial Genome of Tabanidae (Diptera: Tabanidae) Reveals the Present Status of Tabanidae Classification
Source: Insects. 2022 Aug 3;13(8):695. doi: 10.3390/insects13080695 (PMC9408937; doi:10.3390/insects13080695)

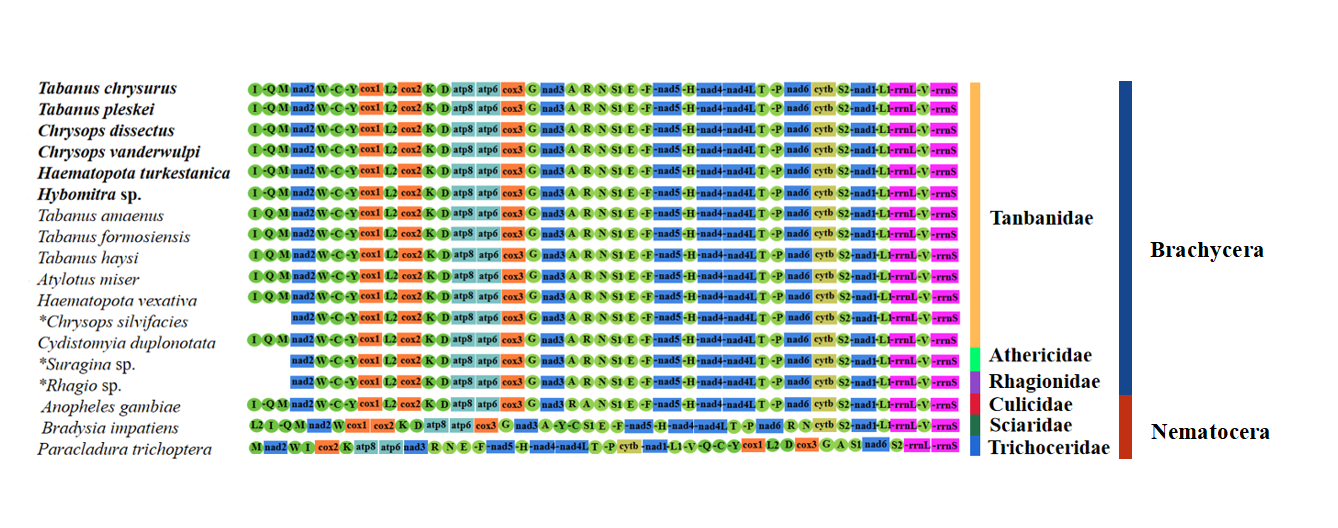

Supplement: Supplementary file 1 [file insects-13-00695-s001.zip › insects-1822810-supplementary/Figure S1 .png]

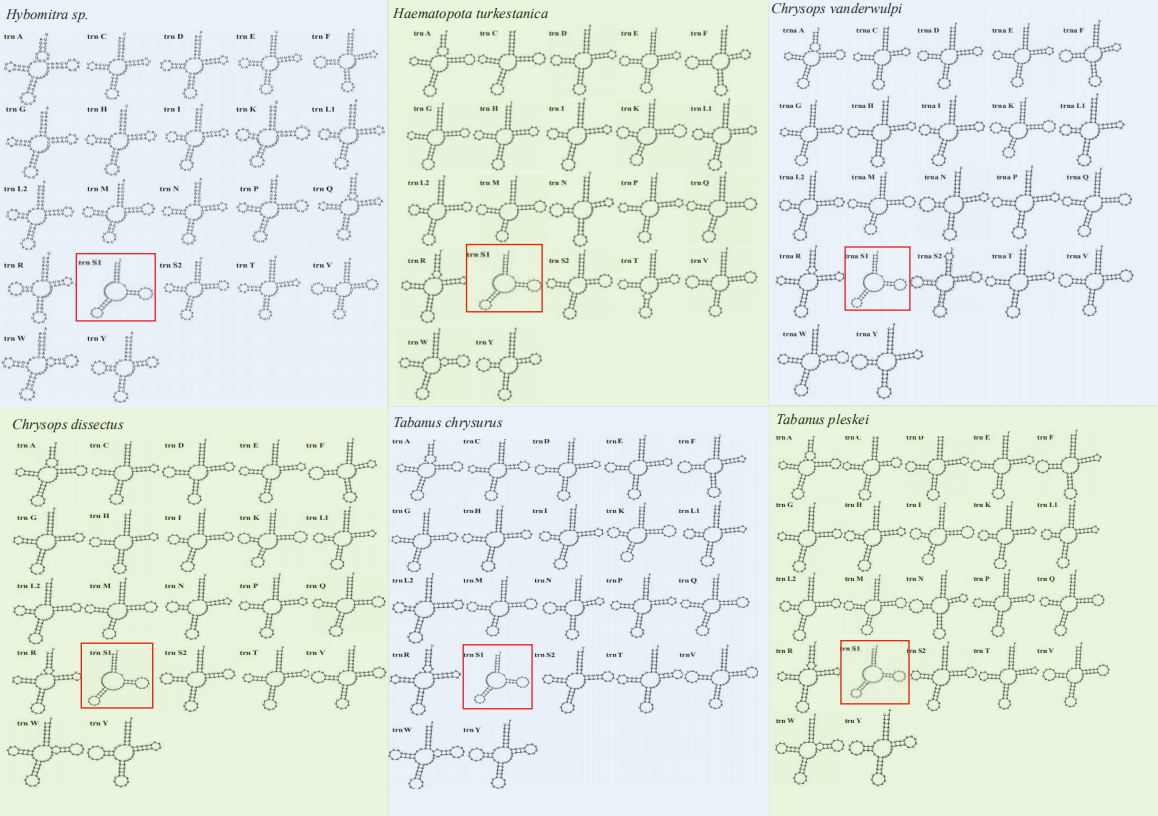

Supplement: Supplementary file 1 [file insects-13-00695-s001.zip › insects-1822810-supplementary/Figure S2 .png]
